# Quackery Rampant

**Published:** 1890-03

**Authors:** R. E. Moss

**Affiliations:** Mexia, Texas


### QUACKERY RAMPANT.

MEXIA, TEXAS, March 3, 1890.

*Editor Daniel's Texas Medical Journal:*

As the Texas State Medical Association is to meet soon, and, as usual, I presume, the subject of the necessity of medical legislation will occupy a part of the time and attention of the members, I beg to contribute my mite of evidence as to the existence of quackery, and the necessity of a law to regulate the practice.

The enclosed letter was written by an eminent (?) *soi disant* gynecologist and specialist. He is a great ignoramus; but many of his sort are practicing under the authority of our great "certificate" law, and there is no remedy at present for the evil.

[THE LETTER.]

——— TEXAS Jany 8, '90.

*Dr.* ————, *Druggist:*

dear Sir I learn that there is a small box of medicine at your drug store for me or sent in your care if so send it out by the bearer. Also I sent some time back after a little bill of drugs and with them a thermomiter and the thermomiter was broken when I receivsd it dont see how it could of got broken in coming from there to me will you please replace one.

Yours truly

——————— M. D.

In connection with the above I give you a new treatment for fecal impaction of the lower colon, as practiced by an ex-President of the Eclectic Medical Association. He was called to see a patient during a violent paroxysm of straining, pain and tenesmus. After making an examination (?), he said he would go home and "read up" before venturing to say what was really the matter,—*as he had not treated any tumors for some time*, and was not "up on tumors." He sent to town, however, to get some ox-gall from the market, with which "to soften the fecal matter." Next day he made a speculum examination, and with a wise shake of the head pronounced it a "cancerous tumor." Hereupon he *injected the mass with carbolic acid*, and left.

A medical friend of mine was called in; after breaking up the mass of hardened feces with a blunt probe, by the free use of warm soap suds, and a pint of sweet oil per rectum, and two ounces of castor oil by the mouth, he was enabled to bring away the "cancerous tumor" in the shape of a gallon or two of the foulest kind of fecal accumulation,—greatly to the relief of the patient, who, for the first time in his life, was able to discriminate, as Dr. Cupples says in his excellent address as Chairman of the Committee on Medical Legislation,—"between the sufficient and insufficient schools of medicine.

This is a fair sample, Doctor, of the class of men annually turned loose,—by the District Examining Boards,—armed with legal authority to kill with impunity, the ignorant and credulous who believe that a "doctor" is a "doctor," no matter where made; and are able to see no difference, except in such cases as the above. Yours, fraternally, R. E. Moss, M. D.

